# Effects of the Mixture of Xylooligosaccharides and Egg White Protein on the Physicochemical Properties, Conformation, and Gel-Forming Ability of *Culter alburnus* Myofibrillar Protein during Multiple Freeze–Thaw Cycles

**DOI:** 10.3390/foods10092007

**Published:** 2021-08-26

**Authors:** Zhongli Zhang, Zhouyi Xiong, Noman Walayat, Jose M. Lorenzo, Asad Nawaz, Hanguo Xiong

**Affiliations:** 1 College of Food Science and Technology, Huazhong Agricultural University, Wuhan 430070, China; zhangzhongli@webmail.hzau.edu.cn; 2 Fisheries Research Institute, Wuhan Academy of Agricultural Sciences, Wuhan 430207, China; 3 Department of Food Science and Engineering, College of Ocean, Zhejiang University of Technology, Hangzhou 310014, China; Noman.rai66@gmail.com; 4 Centro Tecnológico de la Carne de Galicia, Avd. Galicia N° 4, Parque Tecnológico de Galicia, San Cibrao das Viñas, 32900 Ourense, Spain; Jmlorenzo@ceteca.net; 5 Área de Tecnología de los Alimentos, Facultad de Ciencias de Ourense, Universidad de Vigo, 32004 Ourense, Spain; 6 Jiangsu Key Laboratory of Crop Genetics and Physiology, College of Agriculture, Yangzhou University, Yangzhou 225009, China; 007298@yzu.edu.cn

**Keywords:** egg white protein, xylooligosaccharides, myofibrillar protein, freeze–thaw cycles, *Culter alburnus*

## Abstract

This study focuses on the effect of the mixture (XO/EW) of xylooligosaccharides (XO) and egg white protein (EW) on the physicochemical properties, conformation, and gel-forming ability of *Culter alburnus* myofibrillar proteins (MP) during multiple freeze–thaw (FT) cycles. In our methodology, MP samples added with EW, XO, or XO/EW mixture (1%, *v*/*v*) are prepared, and after multiple FT cycles, the XO or XO/EW-treated samples show significant (*p* < 0.05) inhibition on the decrease of sulfhydryl content and the increase of carbonyl content of MP. Compared with EW, XO or XO/EW could delay the increase of surface hydrophobicity and the decline of secondary and tertiary structural properties of MP, indicating that XO or XO/EW could more effectively increase the stability of MP conformation. Meanwhile, XO/EW could more effectively reduce the decrease of gel strength and gel water holding capacity, and the increase in the T_2_ relaxation time of MP gel, confirming that XO/EW could substantially improve the MP gel-forming ability. Analysis of intermolecular interaction force proves that, compared with EW, XO/EW could reduce the content decrease of ionic and hydrogen bonds in MP gel. Overall, XO/EW could improve the stability of MP functional properties over multiple FT cycles. This study provides a new perspective for the potential commercial application of EW as a low-calorie cryoprotectant in aquatic products.

## 1. Introduction

In China, *Culter alburnus* is widely distributed in rivers and lakes, and its commercialization and preservation have attracted increasing attention, due to its delicious taste and balanced nutrition [1]. Nowadays, an increasing amount of *Culter alburnus* is preserved through frozen storage for commercial transportation and long-term nutritional stability. Unfortunately, temperature fluctuation during transportation and in-complete cold chains would negatively affect the quality of aquatic products. Additionally, aquatic products are repeatedly frozen, stored, and thawed during consumption (such as hotels and homes) to facilitate processing, thereby decreasing the functional and nutritional attributes of the final product [2]. Moreover, repeated freezing-thawing cycles will degrade the quality of the product and ultimately reduce its commercial value.

Myofibrillar protein (MP), an essential component of fish protein (about 61%), mainly including myosin, actin, and troponin, plays an essential part in fish muscle composition [3,4]. Basically, the physicochemical properties, conformation, and gel-forming ability of myofibrillar protein are important for the proper gel-formation and texture characteristics of processed fish muscle products, such as fish sausages, fish cakes, and fish balls. However, the oxidation of MP and the growth of ice crystals during freezing will cause the denaturation and irregular aggregation of protein molecules, reduce protein solubility and sulfhydryl content, and increase the content of carbonyl and dityrosine groups [5]. Meanwhile, the surface hydrophobicity, as well as tertiary and secondary structure, will be altered [6]. These changes would alter the intermolecular interaction forces of heat-induced MP gel between protein-protein and protein-water molecules, thus affecting the gel water-holding capacity (WHC) and gel strength. Ultimately, the degeneration of the physicochemical properties, conformation, and gel-forming ability of MP would cause a reduction in the edible value of the product, resulting in undesirable flavors, tenderness loss, or texture deterioration.

To minimize protein denaturation and aggregation, cryoprotectants are usually added in MP to prevent and delay the quality-related changes during freeze–thaw (FT) cycles [2]. The cryoprotectants commonly used in the industry are sucrose and sorbitol, which can affect the flavor of the product, increase extra calories, and adversely affect the quality of the product [1]. Recently, researchers have discovered the potential of some organic acids, protein hydrolysates, and oligosaccharides as cryoprotectants [5,7]. Generally, cryoprotective agents can be divided into two types according to their cryoprotective mechanism for proteins: (i) The first type of cryoprotectants, such as sucrose, xylooligosaccharides, etc., which bind to proteins through their chemical groups by intermolecular interactions, thereby increasing the stability of the protein conformation and delaying the deterioration and aggregation of proteins during long-term freezing or freeze–thaw. Besides, this type of protective agent can also replace the water around the surface of the protein, thereby inhibiting the formation of ice crystals from the water molecules around the protein and reducing the deteriorating effect of ice crystals on the protein. Furthermore, substances, such as carrageenan oligosaccharides and polyphenols can also reduce the oxidative deterioration of MP through their antioxidant effects; (ii) the second type of cryoprotectants, such as egg white protein and soy protein isolate, which can form a molten globular protein when heated to improve the gelling strength of the fish protein gel network through the filling effect. However, most previous studies only focused on the effect of a single substance as a cryoprotectant on MP during frozen storage, such as sucrose, sorbitol, oligosaccharides, and polysaccharides, although their cryoprotective abilities as cryoprotectants can be enhanced when used in combination with other food-based additives, including egg white protein (EW), albumin, polyphenols, etc. [8]. 

EW has become one of the essential additives in aquatic products, due to its wide range of sources, low price, and various properties, which are conducive to the emulsification, foaming, and gelling properties of surimi and seafood products [9]. However, the function of EW protein is limited by the molecular basis of protein structure in the food industry [10]. Several studies have shown that EW can be modified by the thermo-physical or chemical methods (such as stir-mix, heat-induced denaturation, or Maillard reaction) to improve its emulsifying properties, thermal stability, and gelling abilities [10,11,12]. 

Xylooligosaccharides (XO), which are produced by the hydrolysis of xylan through endo-1,4-β-xylanase and cannot be absorbed by the stomach or intestines [13], play an essential role in regulating human health, especially in promoting the growth of bifid-bacteria better than other oligosaccharides [14]. It is worth noting that XO, as a cryoprotectant, has a better protective effect than other sugars, such as sucrose, and does not add extra calories [15]. Bin Zhang et al. [16] demonstrated that XO forms hydrogen bonds with myosin to replace its surrounding water molecules, thereby reducing the negative effect of ice crystals on MP during frozen storage. Furthermore, XO can also interact with myosin and reduce the flexibility and fluctuation of the myosin chain to increase the stability of MP [16].

Despite many studies on the effects of cryoprotectants in protecting freshwater fish during frozen storage, to our knowledge, no study has ever been performed regarding the protective effect of EW and XO mixture on *Culter alburnus* MP during FT cycles. Therefore, the purpose of this study was to evaluate the performance of the XO and EW mixture (XO/EW) in protecting *Culter alburnus* MP during multiple FT cycles by analyzing its impact on the MP physicochemical properties, conformation, and gel-forming abilities. Meanwhile, the individual effect of EW or XO and the combined effect of XO/EW mixture on MP during freeze–thaw cycles were also investigated and compared to enrich our understanding of the properties of XO, EW, and their combination as cryoprotectants. This study provided insights to improve the effectiveness of EW as a low-calorie cryoprotectant.

## 2. Materials and Methods

### 2.1. Materials

Wuhan Academy of Agricultural Sciences Institute (Wuhan, Hubei, China) provided *Culter alburnus* (weight: 2.1 ± 0.3 kg; n = 10) and fresh eggs. The live fish with crushed ice were sent to the laboratory, then immediately stunned by a blow to the head and slaughtered. Next, the fish samples were filleted, minced to surimi, and used for MP preparation. Xylooligosaccharides and other reagents used in this study were purchased from Sinopharm Chemical Reagent Co., Ltd. (Shanghai, China).

### 2.2. Methods

#### 2.2.1. Preparation of the Mixture (XO/EW) of Xylooligosaccharides and Egg White Protein

Egg white protein (EW) was obtained by freeze-drying the egg white, followed by dissolving XO and EW in distilled water at a mass ratio of 1:1 (*w*/*w*). Meanwhile, the final concentration of the XO/EW mixture was 10% (*w*/*v*), and its pH was adjusted to 7.0 with 0.1 mol/L sodium hydroxide and hydrochloric acid. Finally, the mixture was stirred magnetically at 4 °C for 2 h and freeze-dried to obtain the XO/EW product.

#### 2.2.2. Preparation of Myofibrillar Proteins

The preparation of myofibrillar protein extracted from surimi followed the method reported by Zhang et al. [3]. Briefly, surimi (100 g) was homogenized with 500 mL low-salt buffer (0.05 mol/L NaCl, 20 mmol/L PBS, pH = 7.5) using a homogenizer (5000 r/min, 4 °C, 1 min) (XHF-DY, Ningbo Scientz Biotechnology Co, LTD., Ningbo, China), and centrifuged (8000× *g*, 4 °C, 15 min) (AG-22331, Eppendorf, Hamburg, Germany) to separate the precipitate. Next, the precipitate was homogenized with low-salt buffer and centrifuged, and this procedure was repeated twice. Subsequently, the obtained precipitate was homogenized in 4 volumes (m/v) of high-salt buffer (0.6 mol/L NaCl, 20 mmol/L PBS, pH = 7.5) and stored at 4 °C for 12 h to dissolve the MP completely in high salt buffer solution. Finally, the high-salt buffer mixed solution was centrifuged (8000× *g*, 4 °C, 15 min) to separate the supernatant, followed by mixing the supernatant with 10 volumes of (*v*/*v*) 4 °C distilled water and centrifugation immediately. After centrifugation, the obtained precipitate was defined as the MP, and the protein content was determined by the biuret method. The obtained MP content was 87.6 mg/mL.

According to the wet protein content of MP samples, EW, XO, or XO/EW (1%, *w*/*w*) were added separately and mixed uniformly. The MP samples without added EW, XO, or XO/EW were used as the control group.

In this study, 1 FT cycle was defined as freezing the samples at −18 °C for 42 h and then thawing at 25 °C for 6 h. All treatment groups were exposed to 0, 1, 2, 3, and 4 FT cycles.

The unheated MP samples were used to analyze the protein solubility, carbonyl content, sulfhydryl content, dityrosine content, Ca^2+^-ATPase activity, surface hydrophobicity, intrinsic fluorescence intensity, and secondary structural properties.

#### 2.2.3. Preparation of Heat-Induced Gel

The heat-induced gel was prepared, as previously described [17], with a slight modification. Briefly, the MP samples were diluted to 60 mg/mL with PBS buffer (20 mmol/L, NaCl = 0.6 mol/L, pH = 7.5) and transferred to a beaker (height: 2.5 mm, diameter: 3.5 mm). Next, the samples were kept in a 40 °C water bath for 30 min and then in an 80 °C water bath for 30 min. Finally, the gel samples were stored at 4 °C for 12 h before further analysis.

The heat-induced gel was used to test gel strength, water-holding capacity (WHC), T_2_ relaxation time, and intermolecular interaction force.

#### 2.2.4. Protein Solubility

According to a previously reported method [18], each of the MP samples was diluted to ~10 mg/L with 0.6 mol/L NaCl, and then the protein content was determined using the Biuret method [19]. Meanwhile, the sample was centrifuged (10,000× *g*, 15 min, 4 °C) to determine the protein content in the supernatant. Each experimental group was measured three times, and the protein solubility was estimated by the following equation:

(1) $$\mathrm{Protein}\;\mathrm{solubility}\;(\%)=\frac{\mathrm{Protein}\;\mathrm{content}\;\mathrm{after}\;\mathrm{centrifugation}}{\mathrm{Protein}\;\mathrm{content}\;\mathrm{before}\;\mathrm{centrifugation}}\times 100\%$$

#### 2.2.5. Carbonyl Content

As previously reported [20], 2,4-dinitrophenylhydrazine (DNPH) was used to determine the carbonyl content of MP. Briefly, MP was diluted to 5 mg/mL with 0.6 mol/L NaCl, followed by adding 10% (*w*/*v*) trichloroacetic acid (TCA) solution and centrifugation (4 °C, 5000× *g*, 5 min). Next, DNPH (2 g/L, containing 2 mol/L HCl) was added to the precipitate and held at 25 °C in darkness for 1 h. Subsequently, the mixture was precipitated with 20% (*w*/*v*) TCA and centrifuged (4 °C, 5000× *g*, 5 min), followed by washing the precipitate three times with 1 mL of ethanol: ethyl acetate (1:1, *v*/*v*), dissolving the precipitate in 2 mL of guanidine hydrochloride (6 mol/L guanidine hydrochloride, containing 20 mmol/L PBS, pH = 6.5), and measuring the UV absorbance value at 410 nm. Finally, the carbonyl content was calculated using the absorption coefficient (22,000 mol·L^−1^ cm^−1^), and the results were expressed as nmoL/mg protein.

#### 2.2.6. Sulfhydryl Content

According to a previous method [3], 5,5′-dithiobis (2-nitrobenzoic acid) (DTNB) was used to determine the sulfhydryl content. The absorbance of each sample was measured at 412 nm, and the results were calculated using the molar extinction coefficient 136,000 M^−1^∙cm^−1^/L and expressed as nmoL/mg protein. Each experimental group was measured three times.

#### 2.2.7. Dityrosine Content

The dityrosine content of MP was determined as reported by Nikoo, Benjakul, and Xu [21] with slight modifications. Briefly, each of the MP sample solutions was diluted to 5 mg/mL, and the fluorescence intensity of each sample was measured using a fluorescence spectrophotometer (F-4600, Hitachi High Technologies Corporation of Japan, Tokyo, Japan). The excitation wavelength was 325 nm, the emission wavelength was 420 nm, and the slit width was 10 nm. The results were expressed as an arbitrary unit (a.u.). Each experimental group was measured three times.

#### 2.2.8. Ca^2+^-ATPase Activity

Ca^2+^-ATPase activity was determined by following the method of Chanarat and Benjakul [22]. Briefly, NaCl (0.6 mol/L) was used to adjust the MP concentration to 5 mg/mL, followed by mixing 1 mL MP (5 mg/mL) with 1 mL CaCl_2_ (0.1 mol/L), 0.6 mL maleic acid (5 mol/L, pH = 7.0) and 7.4 mL distilled water. After adding 0.5 mL of adenosine 5-triphosphate (ATP) solution (containing 20 mmol/L Tris-HCl, pH = 7.0), the reaction was held at 25 °C for 10 min, and then terminated by adding 5 mL TCA (15%, *m*/*v*). After removing the insoluble matter by centrifugation (3500× *g*, 5 min), the inorganic phosphorus content in the supernatant was measured using the method of Fiske and Subbarow [23]. Ca^2+^-ATPase activity was expressed as μmol/mg/min.

#### 2.2.9. Surface Hydrophobicity (S_0_)

Surface hydrophobicity was analyzed by using 1-anilino-8-naphthalenesulfonate (ANS) as the fluorescence probe, as previously reported [3]. Briefly, MP was diluted to 0.2, 0.3, 0.5, and 1 mg/mL, followed by mixing 5 mL diluted MP solution with 20 μL ANS solution (2 mmol/L, containing 0.2 mol/L PBS, pH = 7.5) and reaction in the dark at 25 °C for 30 min. Next, the fluorescence intensity was measured using a fluorescence spectrophotometer at the emission wavelength of 470 nm, the excitation wavelength of 390 nm, and the slit width of 10 nm. Each group was measured three times. The linear slope between fluorescence intensity and protein concentration was calculated and expressed as the MP surface hydrophobicity.

#### 2.2.10. Intrinsic Fluorescence Intensity

The intrinsic fluorescence intensity (IFI) of the MP samples was analyzed using a previously reported method [18]. Briefly, MP samples were diluted to 1 mg/mL using 0.6 mol/L NaCl, and their intrinsic fluorescence intensity was determined using a fluorescence spectrophotometer at the emission wavelength of 300–400 nm, excitation wavelength of 295 nm, and scanning speed of 1200 nm/min. 

#### 2.2.11. Secondary Structure

As previously reported [18], changes in the MP secondary structure were monitored by Circular dichroism (CD) spectroscopy (J-1500-150, JASCO corporation, Tokyo, Japan). Briefly, MP samples were diluted to 0.5 mg/mL with 0.6 mol/L NaCl, and the samples were measured by CD spectroscopy in the wavelength range of 200–250 nm, 200 nm/min speed, 0.1 cm quartz cell, and 1 nm width. Finally, the secondary structure was calculated using Young’s equation.

#### 2.2.12. Gel Strength

The gel strength test was performed using the Texture Analyzer (TA. XT. Plus, A.XT2, Stable Micro System, London, UK), as previously reported [3]. In the test, the spherical probe (P/0.25 s) was used, and the parameters for the texture analyzer were set as follows: 1 mm/s of penetration rate, 10 mm of test depth, 5 g of trigger force, 1 mm/s of speed before and after the test, and 10 mm of return distance. All samples were analyzed ten times, and the gel strength was estimated by the following equation:

(2)  Gel strength (g∗cm)= breaking force (g) ∗ deformation (cm)

#### 2.2.13. Water-Holding Capacity

The determination of WHC followed a previously reported method [17]. Briefly, the heat-induced MP sample (1 g) was centrifuged (10,000× *g*, 10 min, 4 °C). Then, the WHC was calculated by the percentage between the weight of the sample after centrifugation (after water removal) and the weight before centrifugation. Each experimental group was measured three times. The WHC was estimated by the following equation:

(3) $$\mathrm{WHC}\;(\%)=\frac{\mathrm{Wb}-\mathrm{Wa}}{\mathrm{Wc}-\mathrm{Wa}}\times 100\%$$

where Wa is the weight of the centrifuge tube; Wb is the weight of the centrifuge tube and the heat-induced MP gel, and Wc is the wight of the centrifuge tube and remaining sample (after water removal)

#### 2.2.14. T_2_ Relaxation Time Analysis

According to a previously reported method [24], the T_2_ relaxation time of the heat-induced MP gel was measured to analyze the interaction forces between protein-water molecules in MP gel. Briefly, ~2 g gel was placed into a tube (1 cm in diameter), and the transverse relaxation time (T_2_) was measured using an LF-NMR analyzer (Niumag Electric Company, Shanghai, China; magnetic field intensity 0.5 T, resonance frequency 125 kHz). The sparameters were set as follows: Echo time (TE) = 0.4 ms, TW = 4000 ms, PRG = 2, NS = 8, and NECH = 15,000.

#### 2.2.15. Intermolecular Interaction Force

The intermolecular interaction force was determined according to the method of PerezMateos, Lourenco, Montero, and Borderias [25]. Briefly, four different sample solutions were prepared, and pH adjustment was performed using 0.1 mol/L sodium hydroxide and hydrochloric acid.

S_A_ is the 0.05 mol/L NaCl, pH = 7.0; S_B_ is the 0.6 mol/L NaCl, pH = 7.0; S_C_ is the 1.5 mol/L Urea + 0.6 mol/L NaCl, pH = 7.0; and S_D_ is the 8 mol/L Urea + 0.6 mol/L NaCl, pH = 7.0; 

Next, 1 g MP gel sample was weighed and fully dissolved in 9 mL solutions (S_A_, S_B_, S_C_, and S_D_, respectively) using a homogenizer (5000 r/min, 1 min, 4 °C), followed by keeping the different samples at 4 °C for 1 h and then centrifugation (10,000× *g*, 15 min, 4 °C). Finally, the protein content in the supernatant was determined using the Lowry method [26]. The intermolecular interaction forces were calculated using the protein content of various solution supernatants, and the results were presented as mg/mL soluble protein. Each experimental group was measured three times.

Ionic bond = S_B_ − S_A_ (4)

Hydrogen bond = S_C_ − S_B_ (5)

Hydrophobic interaction = S_D_ − S_C_ (6)

#### 2.2.16. Statistical Analysis

SPSS (IBM Corp, Armonk, NY, USA) was used to analyze all data by variance one-way analysis and Duncan’s multiple comparison test (significant analysis determined at *p* < 0.05). Origin 8.0 software (Origin Lab Inc., Northampton, MA, USA) was used to generate the figures. The results were expressed as mean ± SD.

## 3. Results and Discussion

### 3.1. Protein Solubility

As reported by earlier researchers [18], protein solubility affects the gel-forming ability and emulsifying properties of MP. In Table 1, it was shown that, with increasing FT cycles, all the four groups showed a varying degree of decrease in solubility. After the second FT cycle, the four groups showed a significant (*p* < 0.05) difference in protein solubility, with the highest value (77.49%) for the XO group, followed by the XO/EW group (65.91%). Then, the EW group (54.45%), and the control group had the lowest value (45.11%). However, after the fourth FT cycle, the protein solubility showed no significant (*p* < 0.05) difference between the EW group (37.14%) and the control group (40.43%), but was significantly (*p* > 0.05) higher in the XO groups (58.41%) and XO/EW (47.87%) than in the control group. The decrease of protein solubility could be due to the unfolding of protein molecules, the increase of protein-water intermolecular interactions, and the irregular aggregation of MP [3]. Collectively, adding EW, XO, or XO/EW could delay the decrease of MP solubility, but XO and EW/XO showed better performance in this respect and could protect protein-water intermolecular interactions.

### 3.2. Carbonyl Content

The formation of carbonyl groups is always accompanied by MP denaturation and aggregation. Meanwhile, the formation of carbonyl groups could influence the meat product quality, such as texture, flavor, and nutritional value. Therefore, the carbonyl content is an essential indicator for the oxidation of MP and meat products [1]. In Table 1, the carbonyl content was seen to increase significantly (*p* < 0.05) in all four groups, especially the control group. After the second FT cycle, the carbonyl content was significantly (*p* < 0.05) lower in the XO group (2.67 nmol/mg) than in the XO/EW group (3.68 nmol/mg), but both of their values were significantly (*p* < 0.05) lower than the value of the control group (6.19 nmol/mg). Meanwhile, the value was not significantly (*p* > 0.05) different between the EW group (5.14 nmol/mg) and the control group (6.19 nmol/mg). Therefore, compared with EW, adding XO/EW or XO could reduce the formation of carbonyl groups by inhibiting oxidation during FT cycles. 

### 3.3. Sulfhydryl Content

MP is rich in sulfhydryl groups, which can be easily oxidized into disulfide bonds during frozen storage or FT cycles [3]. In Table 1, it was shown that, after the second FT cycle, the sulfhydryl content was significantly (*p* < 0.05) higher in the XO group (17.89 nmol/mg) than in XO/EW group (14.72 nmol/mg), but both of their values were significantly higher than the value of the EW group (11.60 nmol/mg) and the control group (10.09 nmol/mg). Changes in sulfhydryl content indicated that adding XO or XO/EW could reduce the oxidation of MP, which agreed with the results of carbonyl group content. Furthermore, during MP alterations, polypeptide chains in MP were within or between cross-linked by disulfide covalent bonds, resulting in irregular aggregation of MP [27]. These results indicated that, compared with EW, XO/EW could prevent the denaturation and aggregation of MP, which may be related to the intermolecular interaction force of MP during FT cycles.

### 3.4. Dityrosine Content

The dityrosine formed by C-C coupling after oxidation of L-tyrosine is a fluorescent macromolecular amino acid and serves as a protein oxidation marker, resulting in inter-/intra-molecular cross-linking of proteins [28]. After multiple FT cycles, the dityrosine content showed an increase in all the samples, indicating that FT cycles could aggravate the irregular aggregation of MP. The four groups showed a significant (*p* < 0.05) difference in dityrosine content, with a gradual increase in the groups of XO, XO/EW, EW, and the control, which was 2.55, 3.59, 4.76, and 5.34 a.u., respectively. MP oxidation induces the production of hydroperoxides, leading to the gradual formation of carbonyl groups, followed by the degradation of sulfhydryl groups and the formation of dityrosine [29]. Based on a previous report [30], it can be inferred that, compared with EW, the XO contained in the XO/EW mixture could increase the content of hydroxyl groups and protect the interaction forces between MP molecules, thus inhibiting the interaction of carbon-amides and reducing the formation of dityrosine.

### 3.5. Ca^2+^-ATPase Activity

The Ca^2+^-ATPase activity can express the integrity of MP [30]. In Table 1, the Ca^2+^-ATPase activity was seen to decrease significantly (*p* < 0.05) in the four experimental groups after multiple FT cycles. At the second FT cycle, Ca^2+^-ATPase activity had no significant (*p* > 0.05) difference between the control group (0.0712 μmol/mg/min) and the EW group (0.0834 μmol/mg/min), but the two values were significantly (*p* < 0.05) lower than the values of the XO group (0.1235 μmol/mg/min) and the XO/EW group (0.0996 μmol/mg/min). According to a previous study [31], changes in the MP tertiary structure could cause a decline in Ca^2+^-ATPase activity, attributed to the oxidative modification of myosin and alterations in protein-protein macromolecule interactions. Additionally, the myosin head is the catalytic region responsible for Ca^2+^-ATPase activity and rich in sulfhydryl groups [32]. Therefore, the changing trend of Ca^2+^-ATPase activity was the same as the sulfhydryl group content during FT cycles. Generally, a cryoprotectant delays the decline of Ca^2+^-ATPase activity by stabilizing the surface water tension of the protein [1]. Furthermore, as reported by Zhang et al. [16], XO can interact with the water molecules around myosin through hydrogen bonding, inferring that the XO contained in EW/XO might play a major role in slowing down the deterioration of MP molecules during FT cycles by inhibiting the formation of ice crystals from the water on the surface of the protein.

### 3.6. Surface Hydrophobicity (S_0_)

**Figure 1:**
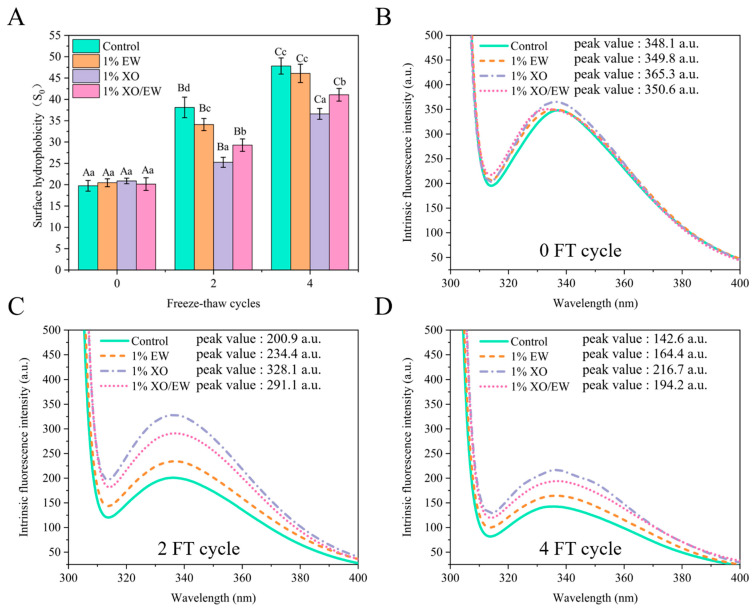
Change of surface hydrophobicity (S_0_ANS) (**A**) in *Culter alburnus* myofibrillar protein added with EW, XO, or XO/EW during freeze–thaw cycles (mean ±SD, n = 3). The control group (Control) was not added with EW, XO, or XO/EW. Capital letters indicate a significant difference (*p* < 0.05) between different freeze–thaw cycles within the same treatment group. Lowercase letters indicate a significant difference (*p* < 0.05) between different treatment groups in the same freeze-thaw cycle. Changes of intrinsic fluorescence intensity in *Culter alburnus* myofibrillar protein added with EW, XO, or XO/EW after 0 (**B**), 2 (**C**), and 4 (**D**) freeze–thaw cycles. The control group (Control) was not added with EW, XO, or XO/EW.

The conformational changes in protein molecules can be evaluated by analyzing surface hydrophobicity (S_0_), which may correspond to the exposure of hydrophobic amino acids [3]. Generally, hydrophobic amino acids are responsible for hydrophobic interaction, hidden in the folding of natural proteins [33]. In Figure 1A, it was shown that, after the fourth FT cycle, the surface hydrophobicity increased significantly (*p* < 0.05) in the four groups, indicating that the FT cycles caused conformation changes and exposed the hydrophobic portions of protein molecules to the surface of MP molecules. It is worth noting that, at the second and fourth FT cycles, the surface hydrophobicity was significantly (*p* < 0.05) lower in the XO group than in the XO/EW group, but both of their values were significantly lower (*p* < 0.05) than the values of the EW and control groups. Hydrophobic interaction between exposed hydrophobic groups might intensify and cause protein aggregation [34]. Compared with EW, XO addition can inhibit conformational changes in MP by interacting with MP [16]. Therefore, the XO contained in XO/EW makes the mixture more effective than adding EW alone in reducing MP conformational changes and delaying irregular MP aggregation.

### 3.7. Intrinsic Fluorescence Intensity

Generally, tryptophan exists in natural proteins and is sensitive to the polar environment [18]. However, some factors may cause the unfolding of MP conformation, exposing tryptophan residues to a polar hydrophilic environment, and reducing intrinsic fluorescence intensity (IFI). As shown in Figure 1B,D, after the fourth FT cycle, the four groups showed a varying degree of IFI decrease, probably due to the exposure of Trp residues to the polar environment and the unfolding of protein molecules [35]. After the second FT cycle, the peak IFI showed a gradual increase, which was 200.9, 234.4, 291.1, and 328.1 for the groups of control, EW, XO/EW, and XO, respectively. Moreover, myosin is the main MP component, which plays an essential role in forming the gel network and has abundant Trp residues in its head and rod regions [36]. Furthermore, as stated by earlier researchers [16], XO can enhance the consistency and cooperativity of peptide chains in myosin and reduce the total interaction energy between myosin and water molecules. Meanwhile, XO can replace the water molecules around the surface of myosin, thereby stabilizing the conformation of myosin during frozen storage or FT cycles, which may explain why XO and XO/EW could delay the reduction of IFI more effectively.

The IFI results agreed well with the results of surface hydrophobicity (Figure 1A), the sulfhydryl content, and Ca^2+^-ATPase activity (Table 1), indicating that, compared with EW, XO/EW was more effective in delaying conformational changes and improving the gel-forming ability of MP. Additionally, protein aggregation was also shown to increase the steric hindrance and cause IFI to decline [37]. Moreover, the functional properties of MP could be affected by changes in the tertiary structure and intermolecular interaction forces, leading to ice crystal growth and protein oxidation [30]. Furthermore, XO can inhibit the growth of ice crystals by forming electrostatic interactions with free water molecules [38]. Therefore, XO/EW can enhance the hydrogen and ionic interaction between MP.

### 3.8. Circular Dichroism

**Figure 2:**
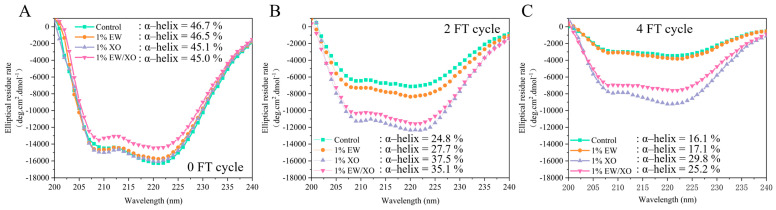
Changes of Far-UV in *Culter alburnus* myofibrillar protein added with EW, XO, or XO/EW after 0 (**A**), 2 (**B**), and 4 (**C**) freeze-thaw cycles. The control group (Control) was not added with EW, XO, or XO/EW.

Circular dichroism spectroscopy can detect changes in the protein secondary structure, which is related to the stability of MP and is influenced by the polypeptide backbone structure and amide region [39]. As shown in Figure 2, after the fourth FT cycle, all four groups had two small peaks at 208 nm and 222 nm in the far-UV spectra, corresponding to changes in α-helical structure [40]. α-helix occupied 90–91% of the secondary structure content of myosin, and these two characteristic peaks are related to the changes of myosin and the gel-forming ability of MP [17]. All four groups showed a decrease in α-helix content, indicating a reduction in the functional properties of MP during multiple FT cycles. At the 0 FT cycle, far-UV spectra were almost similar in the four groups, but showed a varying degree of reduction after the second FT cycles, with an α-helix decrease of 21.9% in the control group, 18.8% in the EW group, 9.9% in the XO/EW group, and 7.6% in the XO groups. Generally, the weak hydrogen bond between the amide-NH and C = O groups is considered the main force for stabilizing the α-helical conformation, which is related to the loss of α-helix content [41]. Oxidation can weaken the hydrogen bond interaction between amino acid side chains and affect the secondary structure of MP [3]. Additionally, oxidation can form disulfide bonds and carbonyl groups, which could affect the conformational properties of MP molecules, indicating the importance of inhibiting MP oxidation during the multiple FT cycles. According to previous reports, XO has antioxidant capacity and can enhance the stability of protein molecules during temperature fluctuations [16,42]. Collectively, the XO contained in XO/EW could effectively maintain the stability of the second structure during FT cycles by interacting with MP, thereby reducing the change of intermolecular interaction force, and delaying the exposure of hydrophobic residues caused by conformational changes.

### 3.9. Gel Strength

Gel strength is an important index to characterize the heat-induced gel-forming ability of MP [3]. The formation and quality of the heat-induced MP gel are determined by the following two steps [3]: (i) Heat induces the orderly unfolding of proteins and the exposure of functional groups, such as sulfhydryl groups, and (ii) MP molecules are cross-linked in the gel system by binding functional groups. Thus, during multiple FT cycles, the sulfhydryl group content was reduced (Table 1), the tertiary structure was unfolded, and the surface hydrophobicity increased (Figure 1), which could be the major factors for reducing gel-forming ability [3].

**Figure 3:**
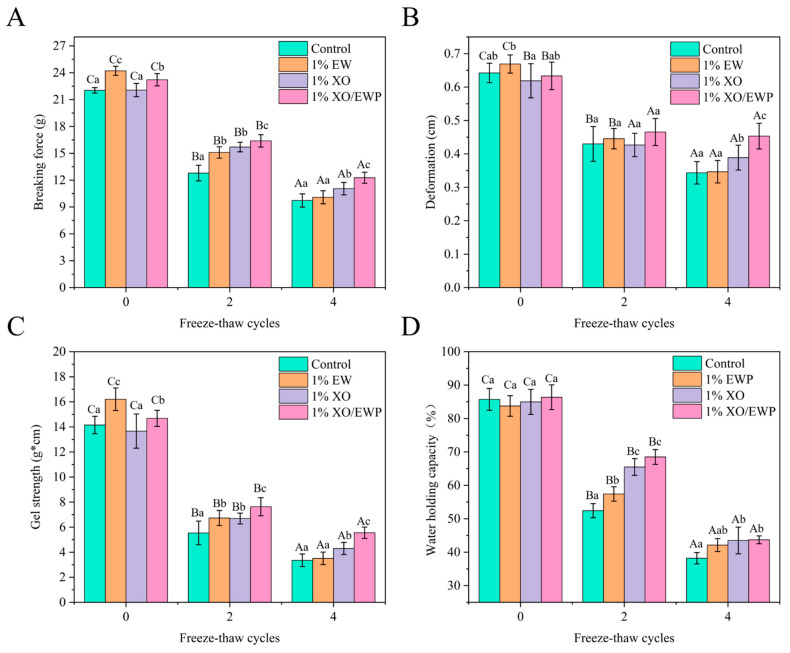
Changes of breaking force (**A**), deformation (**B**), gel strength (**C**), and water-holding capacity (**D**) in the heat-induced gel of *Culter alburnus* myofibrillar protein added with EW, OX, or XO/EW during freeze–thaw cycles (mean ± SD, n = 10). The control group (Control) was not added with EW, XO, or XO/EW. Capital letters indicate a significant difference (*p* < 0.05) between the different freeze–thaw cycles within the same treatment group. Lowercase letters indicate a significant difference (*p* < 0.05) between different treatment groups in the same freeze-thaw cycle.

In Figure 3, at the 0 FT cycle, the breaking force and gel strength were seen to be significantly (*p* < 0.05) higher in the EW and XO/EW groups than in the control and XO groups, indicating that adding EW can increase the gel strength of MP. After the second FT cycle, the breaking force and gel strength were significantly (*p* < 0.05) higher in the XO/EW group than in the EW or XO group, indicating that XO/EW could better improve the gel-forming ability of MP. Meanwhile, the two values showed no significant (*p* < 0.05) difference between XO and EW groups, suggesting the despite different mechanisms of XO and EW for inhibiting the decrease of the gel-forming ability of MP, but the effect is similar. Generally, XO inhibits the deterioration of functional properties and conformation by combining with MP, thereby retarding the degradation of the gel-formation ability of MP [38]. However, unlike XO, EW can improve the gel quality and strength by filling in the network [10]. Thus, the main role of XO in the XO/EW mixture during the FT cycles can be inferred to increase the stability of MP and delay the decline of the gel-forming ability of MP. Meanwhile, the main function of EW in the XO/EW mixture can be assumed to improve the gel strength of MP by filling the gel network during the FT cycles, which may induce changes in MP. Therefore, the mixture of XO and EW is more effective than adding XO or EW alone in protecting MP during multiple FT cycles.

After the fourth FT cycle, the breaking force and gel strength were still significantly (*p* < 0.05) higher in the XO/EW group than in the XO group or the EW group. Meanwhile, the two values were significantly (*p* < 0.05) higher in the XO group than in the EW group, but showed no significant (*p* > 0.05) difference between the EW and the control group. This can be attributed to the stability of XO, because it has a smaller molecular weight, so the effect is better than EW.

### 3.10. Water-Holding Capacity

Besides an indicator for the quality of the heat-induced MP gel, WHC can also be used to reflect the protein-water intermolecular interaction force of the MP gel [3]. As shown in Figure 3D, the showed no significant (*p* > 0.05) difference in the four groups at the 0 FT cycle, but displayed a significant (*p* > 0.05) decrease after the fourth FT cycle, indicating that FT cycles could reduce the MP gel-forming ability and induce the loss of MP gel structure, reflecting the decrease of protein-water intermolecular interactions. 

After the second FT cycle, the WHC was significantly (*p* < 0.05) higher in the XO/EW and XO groups than in the EW group, and it was also significantly (*p* < 0.05) higher in the EW group than in the control group. The results indicate that EW or XO can retard the deterioration of the gel network structure of MP, where EW improved the MP gel network by filling the gel network, while XO inhibited the reduction of MP gel-forming ability by suppressing the decrease of sulfhydryl content and the conformational changes of protein molecules. Additionally, the abundant hydroxyl groups of XO could also immobilize more water molecules through hydrogen bonding, which might explain why WHC was significantly higher in the XO group than in the EW group. The WHC results suggest that the EW contained in the XO/EW mixture can improve the deterioration of the gel network caused by the FT cycle, and the XO contained in the XO/EW mixture can reduce the degradation of MP gel-forming ability by protecting the MP conformational properties and reducing MP aggregation during the FT cycle.

### 3.11. T_2_ Relaxation Time Analysis

**Figure 4:**
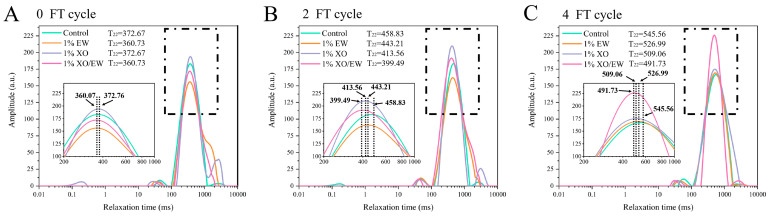
Changes of T_22_ relaxation time in the heat-induced gel of *Culter alburnus* myofibrillar protein added with EW, XO, or XO/EW after 0 (**A**), 2 (**B**), and 4 (**C**) freeze–thaw cycles (mean ± SD, n = 3). The control group (Control) was not added with EW, XO, or XO/EW.

LF-NMR can measure the T_2_ relaxation time to evaluate the restricted water mobility in the MP gel and characterize changes between protein-water intermolecular interaction forces [3]. As shown in Figure 4, each of the four groups showed three peaks after multiple FT cycles. Among them, T_2b_ and T_21_ had the shortest relaxation time (0.01–100 ms), representing the hydrogen protons most closely bound to the MP macromolecular structure. The T_22_ (100–1000 ms) was the major component, representing the immobilized water, which is stabilized by the protein-water molecular interaction in the gel network. The last peak, T_23_ (1000–10,000 ms), represented the filling of free water into the MP network with more mobility [3]. 

As shown in Figure 4, at the 0 FT cycle, the T_22_ relaxation time was higher in the control and XO groups (372.76 ms) than in the EW and XO/EW groups (360.07 ms), proving that the EW could perfect the MP gel network before FT cycles. After second and fourth FT cycles, all the four experimental groups showed an increase in relaxation time, indicating that FT cycles caused the deterioration of the regional water retention capacity of MP gel. These results confirmed the increased surface hydrophobicity, as shown in Figure 1A. Additionally, the irregular aggregation of MP caused the formation of a poor and porous MP gel network, probably due to the interaction of the surface area of the MP gel with water molecules [43]. After the second FT cycle, the T_22_ relaxation time increased to 458.83, 443.21, 413.56, and 399.49 ms for the control, EW, XO, and XO/EW group, respectively, indicating that XO/EW was more effective than EW or XO alone at the same concentration in delaying protein-water molecular interaction and improving the quality of heat-induced MP gel. Moreover, as shown in previous studies [44], certain side-chain groups in the protein, such as carbonyl and sulfhydryl groups (Table 1), are important for the gel-forming ability, due to their ability to bind water molecules, which was confirmed by the significant improvement of MP gel-forming ability after adding XO and XO/EW. Consistent with the WHC conclusion, XO/EW had greater potential than EW in protecting the ability of MP gel to bind water molecules and increase the gel-forming ability. Furthermore, because of its superior interactivity with the functional sites of protein molecules, the EW contained in XO/EW can be filled into the gel gap to improve the gel quality.

### 3.12. Intermolecular Interaction Forces

From a microscopic perspective, the intermolecular interaction force plays a key role in developing a three-dimensional and dense gel network with better WHC and functional properties [45]. Hydrophobic interaction mainly affects the formation of the MP gel network. Besides, ionic and hydrogen bonds are vital forces to restrict the water molecules of the gels [46]. 

**Figure 5:**
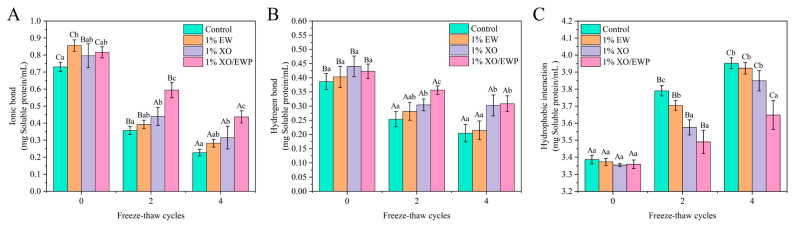
Changes of ionic bond (**A**), hydrogen bond (**B**), and hydrophobic interaction (**C**) in the heat-induced gel of *Culter alburnus* myofibrillar protein added with EW, XO, or XO/EW during freeze–thaw cycles (mean ± SD, n = 3). The control group (Control) was not added with EW, XO, or XO/EW. Capital letters indicate a significant difference (*p* < 0.05) between different freeze–thaw cycles within the same treatment group. Lowercase letters indicate a significant difference (*p* < 0.05) between different treatment groups in the same freeze–thaw cycle.

In Figure 5C, it was shown that the hydrophobic interaction force significantly (*p* < 0.05) increased after the fourth FT cycle. Caused by oxidation, the unorderly unfolding of MP conformational structure and the exposure of functional groups (such as sulfhydryl and hydrophobic groups) caused by oxidation, could increase the protein-protein molecule interactions and affect the formation of the gel matrix during gelation [3]. These results are closely related to the increase of hydrophobicity surface (Figure 1A), reducing IFI (Figure 1C), and the decrease of gel strength (Figure 3C), implicating that the increase in hydrophobic interactions could not only reduce the gelling ability of protein molecules, but also weaken the water-binding ability of MP gel. In the second cycle, the hydrophobic interaction force was not significantly (*p* > 0.05) different from XO and XO/EW groups, and both of their values were significantly (*p* < 0.05) lower than the values of the EW and control groups (Figure 5C). This result indicated that partial replacement of EW with XO is more effective in inhibiting the increase of hydrophobic interactions in MP gels, and adding XO/EW can produce a better result than adding EW or XO alone. 

In Figure 5A,B, the ionic bond and hydrogen bond contents were seen to decrease significantly (*p* < 0.05) after the fourth FT cycle, indicating that multiple FT cycles can affect the protein-water intermolecular interaction forces in each group of MP gels. The ionic bond comes from the repulsive interaction of negative electrostatic force between protein-protein surfaces and the attractive interaction between protein-water molecules [3]. The ice crystal growth and oxidation during FT cycles can alter the MP chemical groups, thus affecting the surface charge distribution and causing the irregular aggregation of MP, leading to the content decrease of ionic bond. The content decrease in hydrogen bond further confirmed that the protein-water interaction force of the MP gel was weakened. Therefore, the content decrease in ionic and hydrogen bonds during FT cycles corresponded to the reduction of water-binding ability and the ultimate increase of water mobility. Additionally, the decline in protein-water interaction is related to the irregular aggregation of MP during gelation [45]. As shown in Figure 5A,B, after the second FT cycle, the ionic and hydrogen bond contents showed no significant (*p* > 0.05) difference except for the EW and control groups, but were significantly (*p* < 0.05) higher in the XO and XO/EW groups than in the control group. Combined with WHC (Figure 3D) and LF-NMR (Figure 4) results, XO/EW was more effective than EW in delaying the content decrease of hydrogen bond and ionic bond in MP gel. Therefore, adding XO/EW could improve the intermolecular interaction force and enhance the functional properties and gelling ability of MP.

## 4. Conclusions

This study evaluated the protective effect of XO/EW on *Culter alburnus* MP during multiple FT cycles. Adding XO/EW in MP was shown to significantly (*p* < 0.05) inhibit the changes in the physicochemical properties of MP caused by multiple FT cycles, thereby reducing the aggregation and degradation of MP. Compared with EW, adding XO or XO/EW was more effective in protecting the functional properties of MP and enhancing the stability of MP conformation during FT cycles. Additionally, adding EW was shown to effectively increase the gel strength of MP and enhance its gel net-work structure by filling the gel network. More importantly, when adding EW or XO alone, XO/EW could significantly (*p* < 0.05) improve the gel strength and WHC of heat-induced MP gel during multiple FT cycles. Moreover, XO/EW could significantly (*p* < 0.05) restrict the decline of ionic and hydrogen-bonding interactions and delay the increase of hydrophobic interactions, thus contributing to maintaining the functional properties of MP gel. Although the deterioration of MP gel quality caused by multiple FT cycles can be inhibited by adding EW or XO alone, XO/EW mixture is more effective than either of them alone, due to its better performance in interaction force, filling the network space, and protecting the MP gel-forming ability. This study provides useful information for combining oligosaccharides and egg white protein to improve the effect of cryoprotectants in protecting MP from the damage caused by multiple FT cycles. Meanwhile, this study also has some limitations, due to its focus on the protective effect of XO/EW on MP in multiple FT cycles. Although MP is the main protein in fish muscle, the effect of XO/EW on other types of proteins in fish muscle is not involved in this study. Moreover, the protective effect of XO/EW on whole fish or its muscle products (such as surimi, etc.) in FT cycles or long-term frozen storage requires further exploration and research.

## Figures and Tables

**Table 1:** Changes of physicochemical properties in *Culter alburnus* myofibrillar protein added with EW, XO, and XO/EW during freeze–thaw cycles.

FT Cycles	Group	Protein Solubility (%)	Carbonyl Content (nmol/mg)	Sulfhydryl Content (nmol/mg)	Dityrosine Content (a.u.)	Ca^2+^-ATPase Activity (μmol/mg/min)
0	Control	91.26 ± 3.44 ^Da^	1.01 ± 0.33 ^Aa^	21.60 ± 0.80 ^Da^	1.52 ± 0.24 ^Aa^	0.1543 ± 0.0078 ^Da^
	1% EW	90.97 ± 2.08 ^Da^	0.98 ± 0.19 ^Aa^	21.33 ± 1.01 ^Da^	1.50 ± 0.21 ^Aa^	0.1678 ± 0.0075 ^Ea^
	1% XO	91.9 ± 2.46 ^Da^	0.97 ± 0.25 ^Aa^	20.93 ± 0.74 ^Da^	1.54 ± 0.24 ^Aa^	0.1637 ± 0.0054 ^Ea^
	1% XO/EW	91.07 ± 3.09 ^Da^	1.01 ± 0.24 ^Aa^	21.74 ± 1.01 ^Da^	1.53 ± 0.26 ^Aa^	0.1620 ± 0.0060 ^Ea^
1	Control	60.67 ± 3.35 ^Ca^	4.26 ± 0.56 ^Bc^	14.68 ± 0.54 ^Ca^	3.59 ± 0.32 ^Bb^	0.0918 ± 0.0094 ^Ca^
	1% EW	69.24 ± 3.08 ^Cb^	3.01 ± 0.31 ^Bb^	16.30 ± 0.81 ^Ca^	3.16 ± 0.29 ^Bb^	0.1076 ± 0.0063 ^Db^
	1% XO	83.59 ± 4.40 ^Cc^	1.85 ± 0.62 ^Aba^	19.65 ± 0.71 ^Dd^	2.11 ± 0.27 ^Ba^	0.1404 ± 0.0076 ^Dd^
	1% XO/EW	77.99 ± 3.23 ^Cc^	2.33 ± 0.48 ^Bab^	17.94 ± 0.66 ^Dc^	2.51 ± 0.25 ^Ba^	0.1257 ± 0.0072 ^Dc^
2	Control	45.11 ± 3.86 ^Ba^	6.19 ± 1.12 ^Cc^	10.09 ± 0.97 ^Ba^	5.34 ± 0.34 ^Cd^	0.0712 ± 0.0071 ^Ba^
	1% EW	54.45 ± 3.52 ^Bb^	5.14 ± 0.89 ^Cbc^	11.60 ± 0.84 ^Ba^	4.76 ± 0.30 ^Cc^	0.0834 ± 0.0083 ^Ca^
	1% XO	77.49 ± 3.27 ^Cd^	2.67 ± 0.63 ^Ba^	17.89 ± 0.88 ^Cc^	2.55 ± 0.31 ^Ba^	0.1235 ± 0.0082 ^Cc^
	1% XO/EW	65.91 ± 3.54 ^Bc^	3.68 ± 0.38 ^Cab^	14.72 ± 0.58 ^Cb^	3.59 ± 0.29 ^Cb^	0.0996 ± 0.0077 ^Cb^
3	Control	38.53 ± 4.12 ^Aa^	7.56 ± 0.50 ^Dd^	7.07 ± 0.63 ^Aa^	6.73 ± 0.35 ^Dd^	0.0527 ± 0.0069 ^Aa^
	1% EW	43.72 ± 4.65 ^Aab^	6.44 ± 0.33 ^Dc^	8.13 ± 0.44 ^Aa^	6.19 ± 0.28 ^Dc^	0.0673 ± 0.0087 ^Bab^
	1% XO	67.73 ± 3.11 ^Bc^	3.63 ± 0.43 ^Ca^	15.14 ± 0.80 ^Bc^	3.30 ± 0.33 ^Ca^	0.0905 ± 0.0094 ^Bc^
	1% XO/EW	52.81 ± 3.94 ^Ab^	4.92 ± 0.28 ^Db^	11.45 ± 0.63 ^Bb^	4.72 ± 0.02 ^Dd^	0.0812 ± 0.0085 ^Bbc^
4	Control	37.14 ± 4.27 ^Aa^	8.15 ± 0.50 ^Dc^	6.01 ± 0.74 ^Aa^	7.86 ± 0.25 ^Ed^	0.0421 ± 0.0074 ^Aa^
	1% EW	40.43 ± 5.03 ^Aab^	7.60 ± 0.41 ^Ec^	6.69 ± 0.80 ^Aa^	7.34 ± 0.25 ^Ec^	0.0471 ± 0.0076 ^Aab^
	1% XO	58.41 ± 5.61 ^Ac^	5.31 ± 0.58 ^Da^	9.29 ± 0.71 ^Ab^	5.15 ± 0.22 ^Da^	0.0694 ± 0.0074 ^Ac^
	1% XO/EW	47.87 ± 3.97 ^Ab^	6.29 ± 0.37 ^Eb^	8.77 ± 0.55 ^Ab^	6.13 ± 0.02 ^Eb^	0.0607 ± 0.0073 ^Abc^

Note: The control group (Control) was not added with EW, XO, or XO/EW. Capital letters indicate a significant difference (*p* < 0.05) between different freeze–thaw cycles within the same treatment group. Lowercase letters indicate a significant difference (*p* < 0.05) between different treatment groups in the same freeze-thaw cycle.

## Data Availability

Not applicable.
